# Quantifying the Coupled Role of Mie Resonance Order and Geometry in Hot-Electron Photodetection

**DOI:** 10.3390/nano16201312

**Published:** 2026-10-17

**Authors:** Qiuyi Long, Longbo Jiao, Jia Hao, Lili Xie, Yizhi Wu, Weijia Shao, Qingjia Zhou

**Affiliations:** 1 Engineering Research Center of Advanced Functional Materials and Intelligent Sensing & School of Physical Science and Technology, Guangxi Normal University, Guilin 541004, China; qylong@stu.gxnu.edu.cn (Q.L.); jlb810975@stu.gxnu.edu.cn (L.J.); jiahao@stu.gxnu.edu.cn (J.H.); llxie@stu.gxnu.edu.cn (L.X.); 2 School of Physical Science and Technology, Tiangong University, Tianjin 300387, China; wuyizhi@tiangong.edu.cn

**Keywords:** hot electron, photodetection, Mie resonance order, coaxial nanowire

## Abstract

Hot-electron photodetectors (HE-PDs) convert photon-excited hot carriers into photocurrents. Although optical resonances often have multiple orders, how the resonance order influences HE-PD performance remains unclear. Using a coaxial Ag–TiO_2_ nanowire as a model system, we investigate the role of Mie resonance order |*m*| in the Ag–TiO_2_ junction. For coaxial nanowires, the Mie resonance order and the device geometry are intrinsically coupled because higher-order Mie resonance necessarily requires a larger TiO_2_ core. By fixing the Ag shell thickness at 8 nm and varying only the TiO_2_ core radius, we excite pure Mie resonances from the first to the eighth order at an identical wavelength of 900 nm. We find that |*m*| directly determines the spatial distribution of hot-electron generation and indirectly leads to increasing separation between the generation site and the metal–semiconductor interface with a rise in |*m*|. Moreover, the internal quantum efficiency of the designed HE-PD decreases under the combined effect of the device size and |*m*| variation, which is attributed to the increased hot-electron travel distance in the coaxial structure and the reduced transport probability. In contrast, external quantum efficiency, which convolutes photon absorption with internal quantum efficiency, increases with |*m*| and reaches its maximum at the sixth order because of the highest normalized absorption cross section at |*m*| = 6. In addition, both the absorption and external quantum efficiency spectra narrow, and their linewidths decrease as the resonance order increases. Overall, our study highlights the critical role of |*m*| intrinsically coupled with device geometry in HE PD performance, deepening our understanding of resonance order effects in hot-electron devices.

## 1. Introduction

When electromagnetic waves impinge upon metallic nanostructures, massive conduction electrons in metals could be driven by the electric field component, giving rise to a diversity of optical resonances as long as corresponding resonance conditions are satisfied [1]. At resonance wavelengths, light–matter interactions are drastically enhanced, and the energies of incident photons could be significantly absorbed by metals, producing abundant energetic electrons (i.e., hot electrons) [2,3]. Extracting hot electrons through metal–semiconductor Schottky junctions has emerged as a promising route to overcome the bandgap limitation of conventional semiconductor photodetectors, even when the incident photon energies are below the bandgap of the semiconductors. Up to now, numerous hot-electron photodetectors (HE-PDs) based on various optical resonances have been demonstrated to enhance photon absorption in metals [4,5,6,7,8], realizing increased populations of generated hot electrons and boosted quantum yields. However, practical HE-PDs based on identical optical resonances often exhibit distinct quantum efficiencies, even when their optical absorption efficiencies are comparable at the same working wavelengths [9,10,11,12]. This calls for a deeper understanding of the combined effects of optical absorptions and specific device configurations on quantum efficiencies, especially on hot-electron dynamics.

Single-nanowire (SN) configurations, serving as a compact optoelectronic platform, have attracted considerable attention owing to their potential applications in various optoelectronic fields, such as in phototransistors [13,14], photovoltaic cells [15,16] and photodetectors [17,18,19]. For an infinitely long nanowire, the structure itself acts as a resonant cavity that confines optical fields within subwavelength volumes. When the radius is in the subwavelength regime, several resonance modes (e.g., Mie resonances and localized plasmonic modes) can be excited, leading to photon energy depositions in absorbable materials. Efforts to combine hot-electron extractions with plasmonic resonance-induced photon absorptions in SN architectures have paid off [20,21]. Most of the recent studies on hot-electron photodetection have only focused on first-order or zero-order optical resonances. Zhan et al. utilized the first-order plasmon modes in coaxial Ag/ZnO/Ag nanowires to achieve asymmetric optical absorption and high-sensitivity hot-electron photodetection [21]. However, they did not investigate the effect of different orders on the performance of the device. Even studies concerning hot electrons linked to Mie resonance are quite limited [22]. Hooshmand et al. showed that higher-order multipole modes significantly contribute to plasmon coupling in metal [23]. However, they did not find the influence on hot electrical photodetection.

For a subwavelength SN HE-PD supporting Mie resonances, the resonant fields inside the structure can be described by Hankel functions of the form $H_{m}^{\left(1\right)}e^{\mathrm{jm\varphi}}$, where the integer *m* denotes orbital angular momentum [24]. For example, *m* = ±1, ±2, and ±3 correspond to dipole, quadrupole, and hexapole resonances, respectively, each exhibiting distinct spatial distributions of the electromagnetic fields. These different field patterns are associated with different optical absorption profiles in metals, inevitably leading to different spatial distributions of hot-electron generation, which in turn affect the transport probability and ultimately the photoelectric conversion efficiency. To the best of our knowledge, no systematic comparison of the Mie resonance order in single-nanowire hot-electron photodetectors has been reported. This is mainly because different orders operate at different frequencies. Optically, the absorption efficiency varies with frequency due to the dispersion of materials [25,26,27,28]. Electrically, the energy distribution of the photoexcited hot electrons also differs for different frequencies [29,30]. As a result, the populations of hot electrons that can transmit the Schottky barrier cannot be directly compared regarding different |*m*|. Moreover, |*m*| is also coupled with the device size. Overall, quantifying the role of |*m*| in SN HE-PDs is highly desired for establishing a quantitative interrelation among the device configuration, photon absorption, and hot-electron dynamics.

In this work, we take a coaxial Ag–TiO_2_ cylinder as a prototype SN HE-PD to systematically investigate how |*m*| determines optical responses and hot-electron processes simultaneously. It forms a Schottky barrier at the Ag/TiO_2_ interface. TiO_2_ is widely used in hot-electron photodetection [5,31,32]. Importantly, for coaxial nanowires, higher |*m*| necessarily requires a larger TiO_2_ core. Therefore, we actually study the coupled effect of resonance order and geometry size. By fixing the Ag shell thickness at 8 nm and monotonically increasing only the TiO_2_ core radius, Mie resonances are excited from the first to the eighth order at a fixed wavelength of 900 nm. Using optical simulations combined with a probability-based electrical model, we reveal that increasing |*m*| directly modulates the electrical field distribution and indirectly alters the generated hot-electron position. The coupled effect of the resonance order and geometry size reduces the hot-electron transport efficiency, leading to a monotonic decrease in internal quantum efficiency (IQE). This is because, for a fixed radial distance from the generation position of the hot electrons to the Ag–TiO_2_ interface, increasing the TiO_2_ core radius increases the hot-electron transport length even when the Ag shell thickness remains fixed. In contrast, device external quantum efficiency (EQE) at 900 nm generally increases with |*m*| due to enhanced photon absorptions. Our findings elucidate an |*m*|-centered, geometry-coupled relationship among device configurations, optical absorption, and electronic processes in SN HE-PDs. Also, this work offers guidelines to tailor the performance of HE-PDs by tuning their resonance order.

## 2. Structure and Optical Analysis

**Figure 1:**
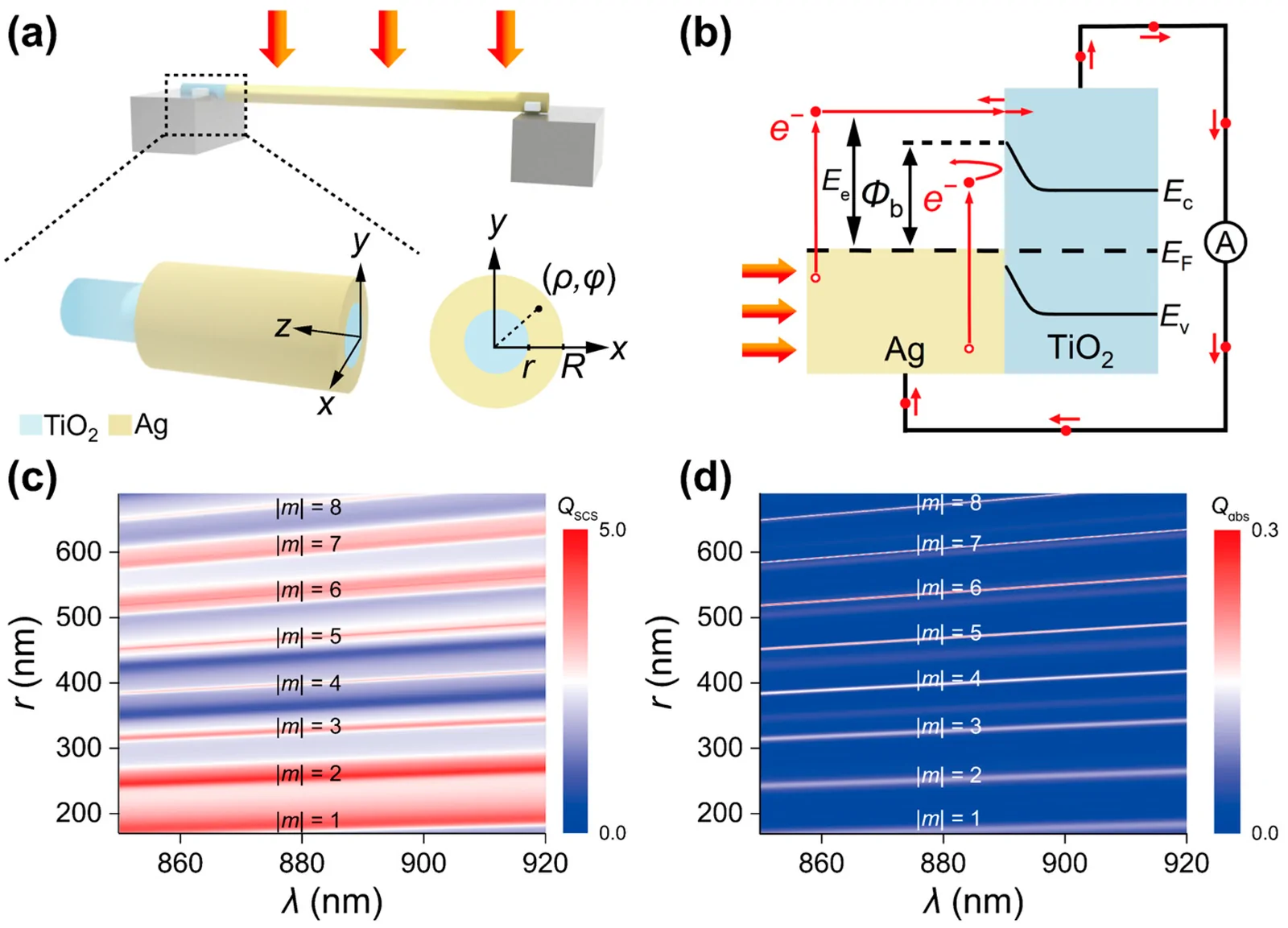
A schematic, an energy band diagram, and the optical responses of the designed single-nanowire hot-electron photodetector (SN HE-PD). (**a**) A schematic of the coaxial Ag–TiO_2_ nanowire. The radii of the TiO_2_ core and nanowire are represented with *r* and *R*, respectively. Polar coordinates (*ρ*, *φ*) are used to determine the position within the Ag shell. (**b**) An energy band diagram of the Ag–TiO_2_ Schottky junction. *E*_c_ (*E*_v_) is the conduction (valence) band edge in the TiO_2_ core. (**c**) The normalized scattering cross section (*Q*_scs_) and (**d**) absorption cross section (*Q*_abs_) depend on *r* and the wavelength (λ) of incident light. The multiband optical responses shown in (**c**,**d**) indicate that the resonance order (|*m*|) at a fixed excitation wavelength can be tuned by *r*.

To quantify the key role of the coupled effects of |*m*| and device geometry in SN HE-PDs, we designed a coaxial Ag–TiO_2_ nanowire whose structure consists of a TiO_2_ core and a Ag shell, as shown in Figure 1a. The thickness of the Ag shell is fixed at 8 nm, which is smaller than the electronic mean free path in Ag [33], so that a substantial fraction of photoexcited hot electrons can diffuse to the Ag–TiO_2_ interface without losing energies. The radius (*r*) of the TiO_2_ core is varied as the primary geometric parameter to control the wavelength of Mie resonances. Figure 1b illustrates that the operations of the designed SN HE-PD rely on three consecutive electronic processes within the Ag–TiO_2_ junction, namely, hot-electron generation, transport, and injection [34,35]. The energy depositions in the Ag shell assisted by Mie resonances induce electronic transitions from occupied levels below the Fermi energy level *E*_F_ to higher unoccupied levels, giving rise to non-equilibrium electrons (i.e., hot electrons). The hot-electron energy exceeding *E*_F_ is denoted by *E*_e_. Once hot electrons arise, they diffuse to the Ag–TiO_2_ interface, experiencing transport losses due to electron–electron and electron–phonon scattering. Upon arriving at the Ag–TiO_2_ interface with a barrier height of *Φ*_b_ (0.91 eV) [1], the above-barrier (*E*_e_ > *Φ*_b_) hot electrons have a chance to be injected into the conduction band of TiO_2_, while the below-barrier (*E*_e_ < *Φ*_b_) hot electrons are blocked by the barrier. The hot electrons entering the TiO_2_ core constitute a measurable photocurrent with the aid of an external circuit. Before comprehensive electrical assessment of the designed SN HE-PD, the optical responses of the coaxial cylinder were studied using the finite-element method implemented in COMSOL Multiphysics 6.1 [36]. We constructed a numerical model in which the designed SN HE-PD is illuminated by electromagnetic radiation whose electric field component oscillates along the *z* direction, and the electric field amplitude (|*E*_0_|) was set to be 1 V/m. This transverse-electric (TE) incident light propagates along the *y* direction. The numerical modeling analysis predicted the wavelength-dependent normalized cross sections of scattering (*Q*_scs_) and absorption (*Q*_abs_), as depicted in Figure 1c and Figure 1d, respectively. The numerical simulation details for *Q*_scs_ and *Q*_abs_ are provided in Section S1 of the Supplementary Materials. Figure 1c shows that *Q*_scs_ is related to *r* and the incident wavelength *λ*. Within the *r* range from 170 nm to 700 nm, eight distinct bands corresponding to eight resonance orders are observed. Similarly, Figure 1d also exhibits eight distinct bands associated with enhanced *Q*_abs_. In general, a clear series of bands provides vital clues regarding excitations of Mie resonances, including the first to the eighth orders. Eight bands have been labeled with corresponding values of |*m*| in Figure 1c,d.

To further confirm that multiband optical responses are due to multiple-order Mie resonances, we analyzed the optical responses of the designed SN HE-PD using Mie theory. For a TE-polarized plane wave propagating along the *y* direction, the electric field outside the coaxial nanowire can be expressed as [37]

(1) $$E_{z}^{I}=\sum_{m=-\infty }^{\infty }\left[A_{m}H_{m}^{\left(1\right)}(k_{1}\rho )+j^{-m}J_{m}(k_{1}\rho )\right]e^{\mathrm{jm\varphi}}$$

where *A_m_* is the *m*th-order scattering coefficient, $H_{m}^{\left(1\right)}$ is the *m*th-order Hankel function of the first kind, and *k*_1_ = *n*_1_*k*_0_ is the wave vector in the background medium, where *k*_0_ = 2π/*λ* is the free-space wave vector and *n*_1_ is the refractive index of the background medium (air in this work). In addition, (*ρ*, *φ*) are the polar coordinates centered at the origin of the coaxial structure. In the Ag shell, the electric field can be evaluated by

(2) $$E_{z}^{\mathrm{II}}=\sum_{m=-\infty }^{\infty }\left[B_{m}^{+}H_{m}^{\left(1\right)}(k_{2}\rho )+B_{m}^{-}H_{m}^{\left(2\right)}(k_{2}\rho )\right]e^{\mathrm{jm\varphi}}$$

where $B_{m}^{+}$ and $B_{m}^{-}$ are the coefficients of the outward and inward propagating modes, respectively. In addition, *k*_2_ = *n*_2_*k*_0_, and *n*_2_ is the refractive index of Ag. Then the electric field within the core can be written as

(3) $$E_{z}^{\mathrm{III}}=\sum_{m=-\infty }^{\infty }\left[C_{m}J_{m}(k_{3}\rho )\right]e^{\mathrm{jm\varphi}}$$

where *C_m_* are the coefficients of the Bessel function and *k*_3_ = *n*_3_*k*_0_ (*n*_3_ the refractive index of TiO_2_). Using the corresponding boundary conditions, we can solve for the scattering coefficients *A_m_* and then obtain the scattering and absorption cross sections. Details of the derivation are given in Section S1b of the Supplementary Materials.

**Figure 2:**
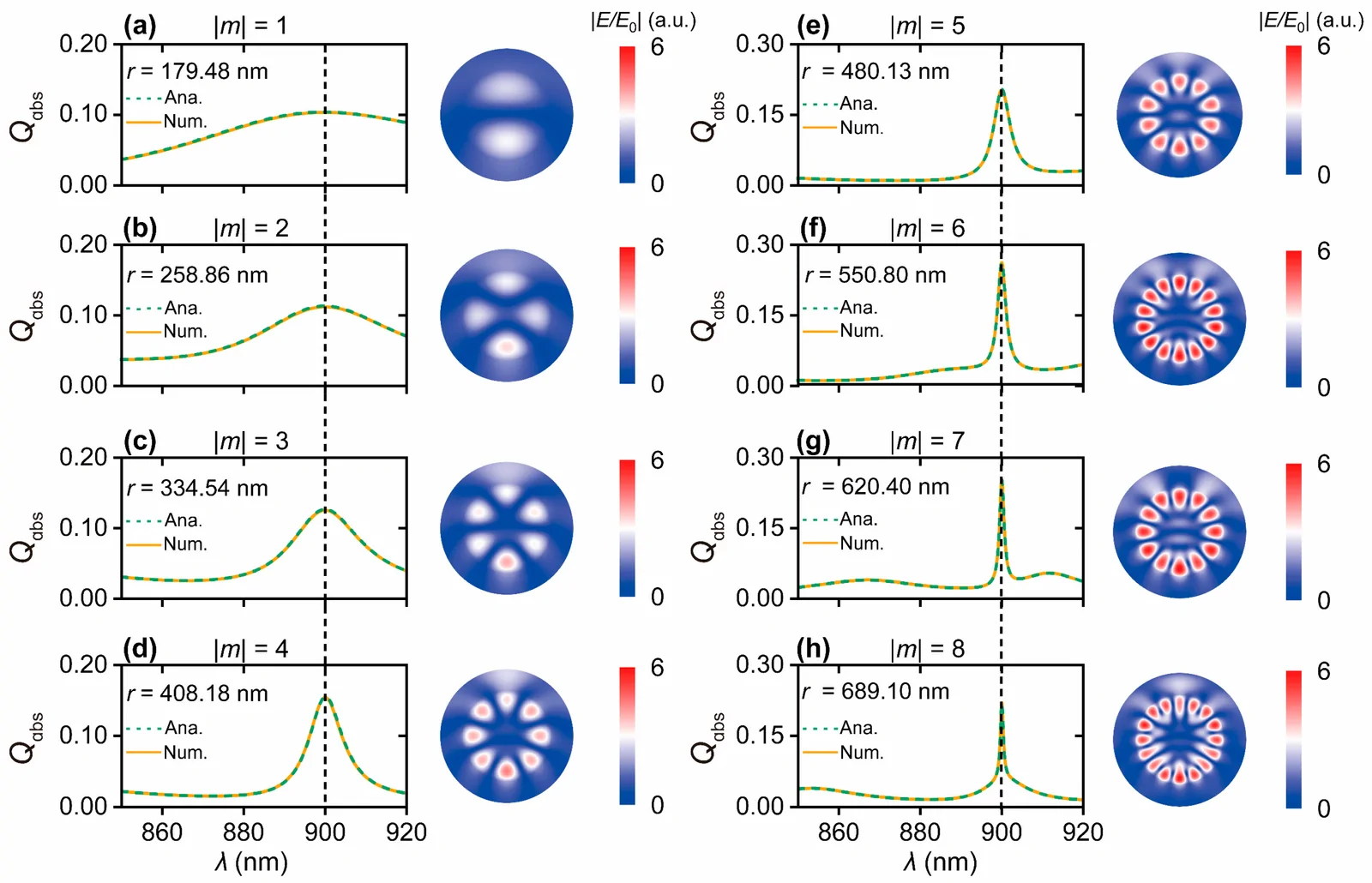
The excitation of Mie resonances with different |*m*| at identical wavelengths of 900 nm by elaborately choosing *r*. The TiO_2_ core radii are (**a**) 179.48 nm, (**b**) 258.86 nm, (**c**) 334.54 nm, (**d**) 408.18 nm, (**e**) 480.13 nm, (**f**) 550.80 nm, (**g**) 620.40 nm, and (**h**) 689.10 nm, corresponding to the 1st (|*m*| = 1) through 8th (|*m*| = 8) Mie resonance orders, respectively. In each subfigure, the left panel shows the numerical and analytical *Q*_abs_ as a function of wavelength *λ*, and the right panel displays the normalized electric field distribution (|*E*|/|*E*_0_|) at 900 nm.

Aiming at excitation of Mie resonances at 900 nm with variable |*m*|, we chose eight *r* values of 179.48 nm, 258.86 nm, 334.54 nm, 408.18 nm, 480.13 nm, 550.80 nm, 620.40 nm, and 689.10 nm, as shown in the left panels of Figure 2a–Figure 2h, respectively. It is found that photon absorption at 900 nm is enhanced. To validate the calculation, the analytical wavelength-dependent *Q*_abs_ (solid curves) is compared to the corresponding simulated results (dashed curves). The analytical solutions agree well with the numerical results, verifying that Mie resonances coupled with device size govern the observed optical responses. The right panels of Figure 2a–h show the |*m*|-related patterns of electric fields (|*E*|) at the resonance wavelength of 900 nm, revealing that the enhanced *Q*_abs_ at 900 nm stems from the confinement of enhanced electric fields (i.e., |*E*/*E*_0_| > 1). Moreover, with increasing |*m*|, the resonance linewidth becomes narrower and the excited field becomes stronger. In the case of Mie resonance excitations, the absorption efficiency is maximum for the sixth order. This is because the sixth-order resonance is closer to the critical coupling condition [38] than that for the other orders. Notably, the resonance of each order was tuned to occur at the same wavelength of 900 nm. This allows us to compare the electrical performance of different orders at a fixed excitation wavelength, eliminating the interference of wavelength shift in optical absorption and hot-electron excitation. Note that, as the resonance order increases, the effect of device size cannot be distinguished from that of |*m*|. Hence, we attribute this to the coupled effect of the resonance order and geometry.

## 3. Electrical Analysis

**Figure 3:**
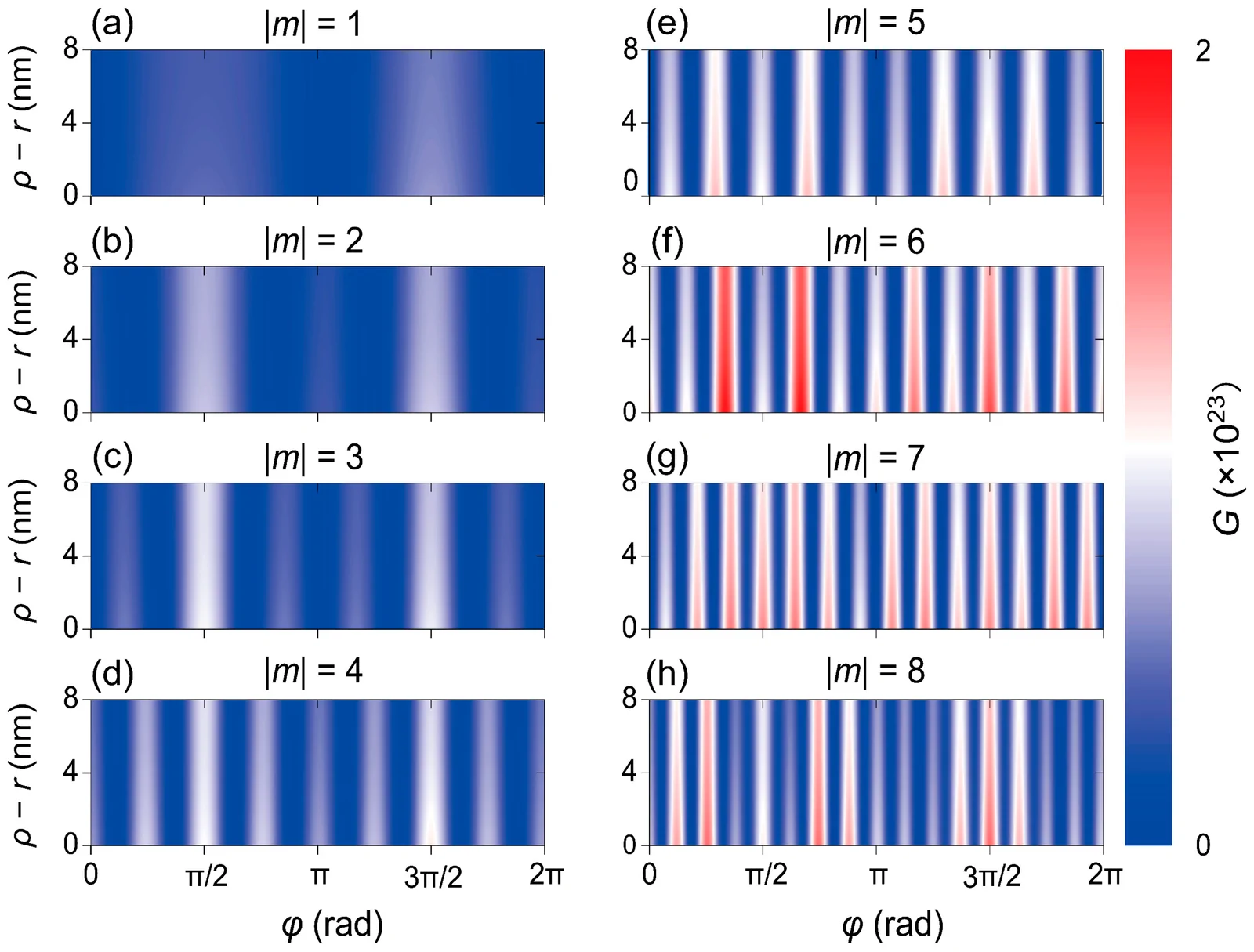
Spatial distributions of the hot-electron generation rate (*G*) for resonance orders from the 1st to the 8th. Panels (**a**–**h**) correspond to the 1st–8th resonance orders, respectively. To clearly display the periodic spatial distributions of *G*, the Ag shell is shown unfolded: the horizontal axis corresponds to the azimuthal angle (*φ*), and the vertical axis (*ρ* − *r*) represents the radial coordinate range occupied by the Ag shell.

After emphasizing the fundamental role of |*m*| and device geometry in optical responses, we demonstrate how |*m*| affects the hot-electron micro processes within the Ag–TiO_2_ junction. Assuming that one absorbed photon generates only one hot electron–hole pair [39,40], the hot-electron generation rate (*G*), which represents the population of generated hot electrons per second, could be written as

(4) $$G=\frac{1}{2\hbar }\varepsilon_{i}|E(\rho ,\varphi ){|}^{2}$$

where *ε*_i_ is the imaginary part of the material permittivity, *E*(*ρ*, *φ*) is the electric field at position (*ρ*, *φ*), and ℏ is the reduced Planck constant. Because the excited field becomes stronger with increasing order, the hot-electron generation rate also increases with the order. In particular, the spatial distribution of the generated hot electrons stems from the mode distribution of the Mie resonances, as shown in Figure 3a–h. In contrast to purely planar counterparts where the hot-electron generation distribution along the metal–semiconductor interface is uniform, the coaxial nanowire device exhibits periodic *G* variation along the Ag–TiO_2_ interface, with the angular period controlled by |*m*|. The radial variation in *G* is slight due to the ultrathin Ag shell. In any case, |*m*| sculpts the hot-electron generation patterns in the Ag shell.

**Figure 4:**
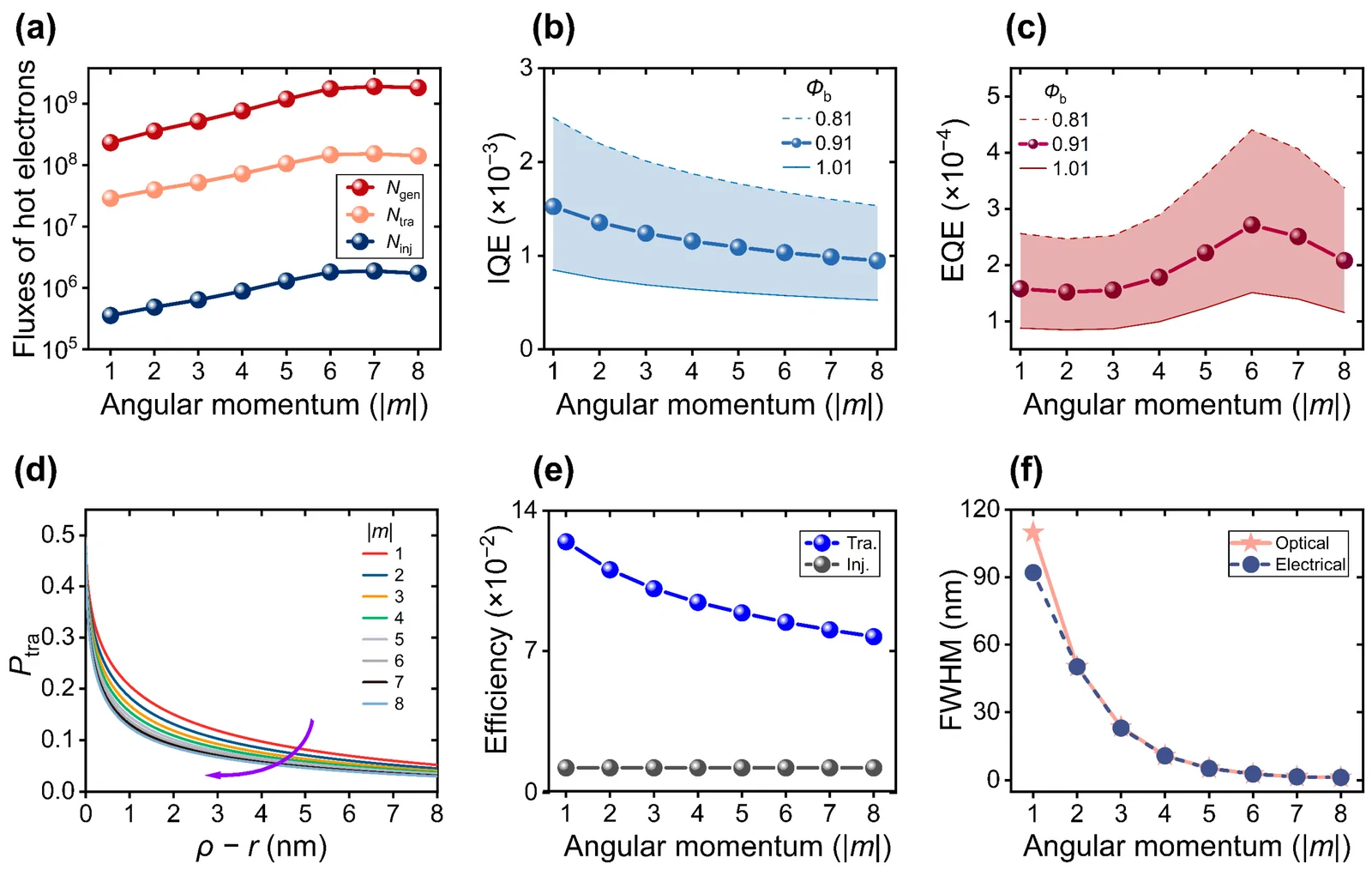
The |*m*|-relevant hot electrons of the coaxial nanowire HE-PD at a resonance wavelength of 900 nm. (**a**) Dependence of hot-electron fluxes of generation, transport, and injection. (**b**) IQE and (**c**) EQE within the barrier height range from 0.81 to 1.01 at 900 nm. (**d**) Transport probability *P*_tra_ as a function of the radial distance from the Ag–TiO_2_ interface (inner wall) to the outer surface of the Ag shell for the 1st to 8th orders. (**e**) Transport and injection efficiencies at 900 nm as functions of the order |*m*|. (**f**) Full widths at half-maximum (FWHMs) of the EQE spectra and the optical absorption efficiency spectra.

Besides |*m*|-related *G* profiles, the fluxes of generated (*N*_gen_), transport (*N*_tra_) and injected (*N*_inj_) hot electrons are also related to |*m*|, as shown in Figure 4a. The definitions of *N*_gen_, *N*_tra_, and *N*_inj_ are presented in Section S2 of the Supplementary Materials. It is found that, when higher-order Mie resonances are excited, *N*_gen_ generally increases with |*m*|, which can be ascribed to the enhanced *Q*_abs_. The maximum flux occurs at the sixth order because *Q*_abs_ is largest for that order. Consequently, *N*_tra_ and *N*_inj_ also generally grow when |*m*| increases. Note that, taking into account errors due to the practical experiment on the material, variable *Φ*_b_ within a relatively broad range from 0.81 eV to 1.01 eV was adopted to calculated *N*_inj_. As shown in Figure 4b, the device internal quantum efficiency (IQE = *N*_inj_/*N*_gen_) [34], which quantitatively describes the hot-electron losses within the Ag–TiO_2_ junction, is cut down with an increase in |*m*|. It seems that enhanced photon absorption at 900 nm cannot bring out an improvement in IQE. On the other hand, the device external quantum efficiency (EQE = *Q*_abs_ × IQE) [34], serving as a main metric to assess device electrical outputs, exhibits a more complex correlation with |*m*|, as shown in Figure 4c. For the variable *Φ*_b_, EQE is boosted when |*m*| increases from 1 to 6 but then reduces when |*m*| further rises. This is because *Q*_abs_ takes a maximum at |*m*| = 6.

To figure out the negative correlation between IQE and |*m*|, the hot-electron transport probability *P*_tra_ along the radial direction was calculated, as shown in Figure 4d. It is reasonable that *P*_tra_ = 0.5 at the Ag–TiO_2_ interface (i.e., *ρ* − *r* = 0 nm). In addition, for fixed |*m*|, *P*_tra_ decreases with increasing radial coordinate *ρ*. For different orders, *r* increases with |*m*|. Thus, at the same *ρ*, higher orders are accompanied by larger *r*, resulting in a smaller *P*_tra_. As |*m*| increases, it becomes more difficult for hot electrons to travel through the same thickness of the Ag shell. This is demonstrated by the transport efficiency (*N*_tra_/*N*_gen_) shown in Figure 4e. The transport efficiency decreases from 0.12 at the first order to 0.08 at the eighth order. Therefore, as |*m*| increases, the device size simultaneously increases. The combined resonance order and geometry effect reduces *P*_tra_, which in turn leads to a decrease in IQE. It presents a tendency of reduction at 900 nm from 0.0015 to 0.0009 when *Φ*_b_ = 0.91 eV. Figure 4e also shows the injection efficiency (*N*_inj_/*N*_tra_) changing with |*m*|. The hot-electron injection efficiency is 0.012, as the injection efficiency is only dependent on the material. A detailed description of hot-electron transport and injection can be found in Section S2 of the Supplementary Materials. Furthermore, as the value of |*m*| increases, the full widths at half-maximum (FWHMs) of both the optical and electrical spectra gradually decrease. The results are shown in Figure 4f.

Within the range of 850 nm to 920 nm, the IQE for the first to eighth orders was also calculated (Section S3 of the Supplementary Materials). The wavelength dependence of IQE and EQE for the first to eighth orders is presented in Section S3 of the Supplementary Materials. For study completeness, we also studied the coaxial nanowire SN HE-PD under transverse-magnetic (TM) polarized excitation. Only four resonance orders (the first, sixth, seventh, and eighth) exhibit strong resonances, and TE polarization is superior to TM polarization regarding hot-electron photoelectric conversion. These results are presented in Section S4 of the Supplementary Materials.

**Figure 5:**
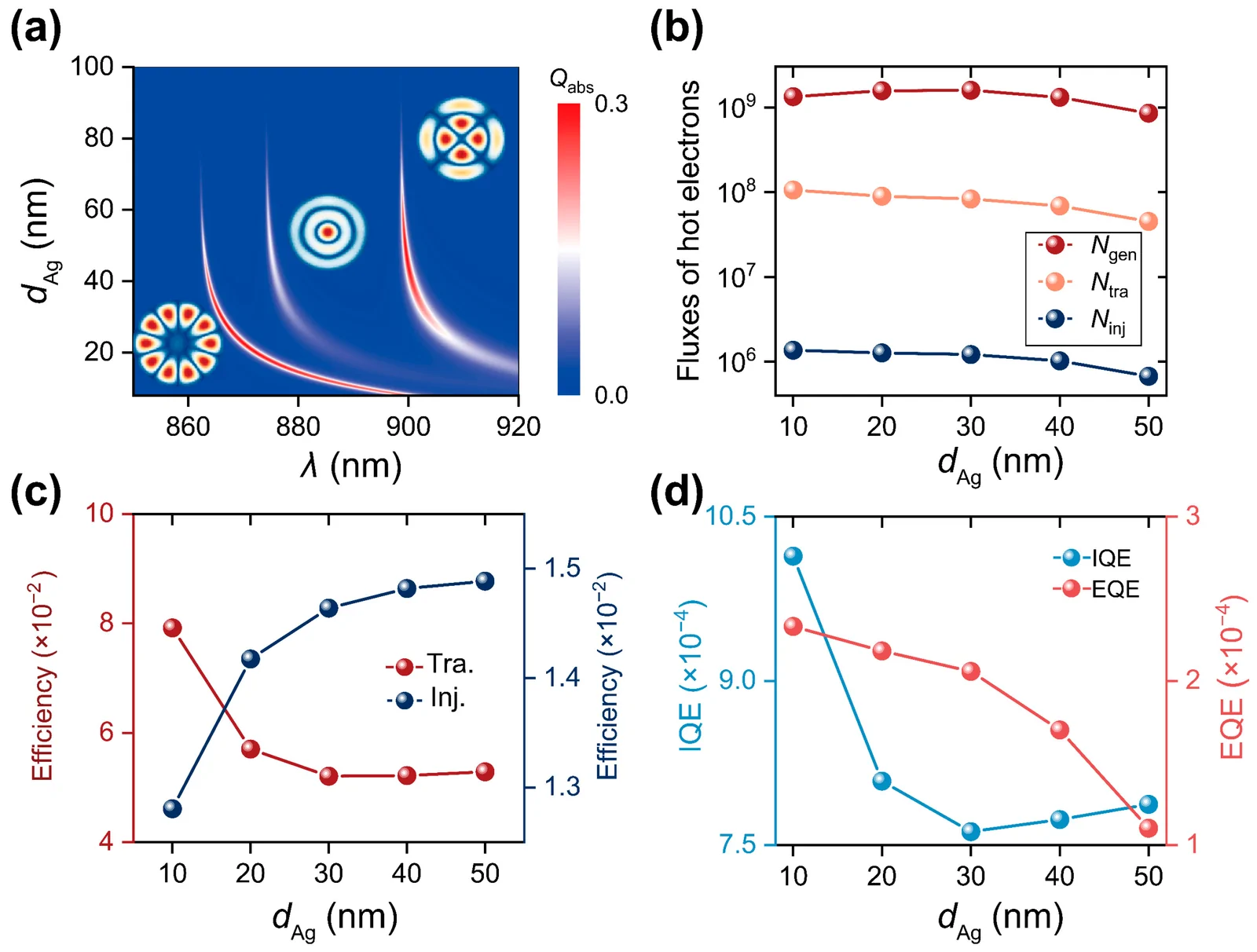
Dependences of optical and electrical performance on variable Ag shell thickness *d*_Ag_. (**a**) The normalized absorption cross section as a function of *λ* and *d*_Ag_. Electric field profiles within three absorption bands are also shown. (**b**) Calculated *N*_gen_, *N*_tra_, and *N*_inj_ at |*m*| = 5 as *d*_Ag_ increases from 10 nm to 50 nm in steps of 10 nm. (**c**) Hot-electron transport and injection efficiencies as a function of *d*_Ag_. (**d**) The calculated device IQE and EQE related to *d*_Ag_.

Finally, we paid attention to the effect of variable Ag shell thickness (*d*_Ag_ = *R* − *r*) on the photoelectric responses of the designed SN HE-PD. Taking the fifth-order Mie resonance as an example, *r* was set to be 480.13 nm when *d*_Ag_ = 8 nm. As depicted in Figure 5a, the Mie resonance wavelength experiences a blue shift with an increase in *d*_Ag_. When *d*_Ag_ increases to 100 nm, fifth-order resonance vanishes due to an excessively thick Ag shell that prevents light from permeating into the TiO_2_ core. Note that, within the studied wavelength range, other higher-order radial modes are excited and exhibit blue shifts as *d*_Ag_ increases. To assess the influence of *d*_Ag_ on device performance at resonance wavelengths, five equally spaced thicknesses (*d*_Ag_ = 10 nm, 20 nm, 30 nm, 40 nm, and 50 nm) were picked for electrical calculations. Figure 5b displays that *N*_gen_ at the resonance wavelength first increases then decreases when the Ag shell is thickened from 10 nm to 50 nm, indicating that a Ag shell with suitable thickness would give rise to maximum photon absorption [41]. Differing from *N*_gen_, both *N*_tra_ and *N*_inj_ at resonance wavelengths reduce when *d*_Ag_ increases from 10 nm to 50 nm, due to the increased hot-electron transport distance. Therefore, hot-electron transport efficiency decreases sharply from 0.08 to 0.058, as shown in Figure 5c. Even though the injection efficiency increases with the thickness of the metal (Figure 5c), the best flux of injection is still at *d*_Ag_ = 10 nm. Figure 5d shows that both IQE and EQE also decrease as the Ag thickness increases beyond 10 nm, primarily due to the rapid decline in transport efficiency. Overall, Ag shell thickness is also a key parameter for tuning the resonance wavelength, but it has a significant influence on optical absorption and hot-electron collection efficiency. Hence, we chose a metal thickness of 8 nanometers to quantify the coupled effect of |*m*| and geometry on hot-electron photodetection.

## 4. Conclusions

To summarize, we have quantified the coupled influence of resonance order |*m*| and corresponding geometry size on hot-electron photodetection in a coaxial Ag–TiO_2_ nanowire supporting Mie resonances. Unlike usual investigations focusing on the dependence of HE PD performance on the working wavelength, we fixed the Ag shell thickness (8 nm) and varied the TiO_2_ core radius to excite first- to eighth-order Mie resonances at 900 nm, guaranteeing feasible evaluations of the underlying correlation between |*m*| and photoelectric responses. The resonance order |*m*| governs photon absorption in the Ag shell and the spatial profile of generated hot electrons, thus modulating the electrical field distribution. The hot-electron transport distance depends not only on the resonance order but also on the core radius. The EQE is highest for the sixth order because of the maximum absorption efficiency. Although the excited field is stronger, the IQE of higher-order modes is lower due to the longer travel distance required for hot electrons to reach the interface. Our work establishes a relevant link between |*m*| and device performance, deepening the understanding of how the resonance order influences hot-electron devices and emphasizing the importance of considering the effect of geometry and resonance order.

## Data Availability

The original contributions presented in this study are included in the article/Supplementary Material. Further inquiries can be directed to the corresponding authors.

## Supplementary Materials

The following supporting information can be downloaded at https://www.mdpi.com/article/10.3390/nano16201312/s1: Figure S1: Device schematic and relevant parameters for evaluating hot-electron transport; Figure S2: Wavelength dependence of IQE for resonance orders 1st to 8th; Figure S3: Wavelength-dependent EQEs for different |*m*|; Figure S4: Normalized absorption cross section change with operating wavelength *λ* and the radius of TiO_2_ *r*; Figure S5: Excitation of TM-polarized Mie resonances of different orders at 900 nm by choosing an appropriate *r*. The left panels show the *Q*_abs_ as a function of *λ*; Figure S6: Wavelength-dependent IQE for the 1st, 6th, 7th, and 8th order resonances under TM polarization.; Figure S7: Wavelength-dependent EQE for the 1st, 6th, 7th, and 8th order resonances under TM polarization. References [42,43,44,45] are cited in the Supplementary Materials.
