# Supplementary material for: Quantifying the Coupled Role of Mie Resonance Order and Geometry in Hot-Electron Photodetection
Source: Nanomaterials (Basel). 2026 Oct 17;16(20):1312. doi: 10.3390/nano16201312 (PMC13707644; doi:10.3390/nano16201312)
Supplement: Supplementary file 1 [file nanomaterials-16-01312-s001.zip › nanomaterials-4597100-supplementary.pdf]

nanomaterials

Supplementary Materials

Quantifying the Coupled Role of Mie Resonance Order and Geometry in Hot-Electron Photodetection

Qiuyi Long[1], Longbo Jiao[1], Jia Hao[1], Lili Xie[1], Yizhi Wu[2], Weijia Shao[1,*] and Qingjia Zhou[1,*]

* Correspondence: wjshao@gxnu.edu.cn (W.S.); qingjiazhou@gxnu.edu.cn (Q.Z.)

S1. Scattering and Absorption Cross Sections

Using a finite-element numerical model, we calculated the normalized scattering cross section (Q_{scs}) that can be written as [1]

$$Q_{scs} = \frac{P_{scs}}{I_{in} \times 2 \times R} \tag{S1a}$$

where I_{in} is the incident intensity and P_{scs} is the total scattering power. We also evaluated the normalized absorption cross section (Q_{abs}) by integrating density of power absorption (Q_{rh}) over the whole Ag shell. Q_{abs} is expressed as [2]

$$Q_{abs} = \frac{\oiiint Q_{rh}\,\mathrm{d}V}{I_{in} \times 2 \times R} \tag{S1b}$$

In specific, Q_{rh} can be calculated with

$$Q_{rh} = \frac{1}{2}\varepsilon_i |E|^2 \omega \tag{S1c}$$

where ω is the angular frequency of the incident light.

For a TE-polarized plane wave propagating along y direction, the total field outside the nanowire ($\rho > R$) can be expressed as [3]

$$E_z^{I} = \sum_{m=-\infty}^{\infty} [A_m H_m^{(1)}(k_1\rho) + j^{-m} J_m(k_1\rho)] e^{jm\varphi} \quad (\rho > R) \tag{S2}$$

where A_m is the scattering coefficient. And the electrical field within the Ag shell ($r < \rho < R$) is

$$E_z^{II} = \sum_{m=-\infty}^{\infty} [B_m^{+} H_m^{(1)}(k_2\rho) + B_m^{-} H_m^{(2)}(k_2\rho)] e^{jm\varphi} \quad (r < \rho < R) \tag{S3}$$

where B_m^{+} and B_m^{-} represent the coefficients of outward and inward propagating waves. The electric field in the TiO_2 core ($\rho < r$) can be written as

$$E_z^{III} = \sum_{m=-\infty}^{\infty} [C_m J_m(k_3\rho)] e^{jm\varphi} \quad (\rho < r) \tag{S4}$$

where C_m is determined coefficient.

According to Maxwell's equations, we could write the magnetic field of each part as

$$H_\varphi^{I} = \frac{k_1}{j\omega\mu_1} \sum_{m=-\infty}^{\infty} [A_m H_m^{(1)\prime}(k_1\rho) + j^{-m} J_m{}'(k_1\rho)] e^{jm\varphi} \quad (\rho > R) \tag{S5}$$

$$H_\varphi^{II} = \frac{k_2}{j\omega\mu_2} \sum_{m=-\infty}^{\infty} [B_m^{+} H_m^{(1)\prime}(k_2\rho) + B_m^{-} H_m^{(2)\prime}(k_2\rho)] e^{jm\varphi} \quad (r < \rho < R) \tag{S6}$$

$$H_\varphi^{\mathrm{III}} = \frac{k_3}{j\omega\mu_3} \sum_{m=-\infty}^{\infty} [C_m J_m'(k_3\rho)] e^{jm\varphi} \quad (\rho < r) \tag{S7}$$

Here $k_i = n_i k_0$ (i = 1, 2, 3). The refractive indexes of air, Ag, and TiO_2 are represented with n_1, n_2, and n_3, while μ_i (i = 1, 2, 3) are the relative permeability of the filling materials, respectively. By applying the boundary conditions at $\rho = R$ and $\rho = r$, we obtained

$$A_m H_m^{(1)}(k_1 R) - B_m^+ H_m^{(1)}(k_2 R) - B_m^- H_m^{(2)}(k_2 R) = -j^{-m} J_m(k_1 R) \tag{S8}$$

$$\frac{k_1}{\mu_1} A_m H_m^{(1)\prime}(k_1 R) - \frac{k_2}{\mu_2}[B_m^+ H_m^{(1)\prime}(k_2 R) + B_m^- H_m^{(2)\prime}(k_2 R)] = -j^{-m} \frac{k_1}{\mu_1} J_m'(k_1 R) \tag{S9}$$

$$B_m^+ H_m^{(1)}(k_2 r) + B_m^- H_m^{(2)}(k_2 r) - C_m J_m(k_3 r) = 0 \tag{S10}$$

$$\frac{k_2}{\mu_2}[B_m^+ H_m^{(1)\prime}(k_2 r) + B_m^- H_m^{(2)\prime}(k_2 r)] - \frac{k_3}{\mu_3} C_m J_m'(k_3 r) = 0 \tag{S11}$$

By solving Equation S7-S10, we can obtain the scattering coefficient

$$A_m = \frac{|B_m|}{|D_m|} \tag{S12}$$

where B_m is

$$\begin{vmatrix} -j^{-m} J_m(k_1 R) & -H_m^{(1)}(k_2 R) & -H_m^{(2)}(k_2 R) & 0 \\ -j^{-m} \frac{k_1}{\mu_1} J_m'(k_1 R) & -\frac{k_2}{\mu_2} H_m^{(1)\prime}(k_2 R) & -\frac{k_2}{\mu_2} H_m^{(2)\prime}(k_2 R) & 0 \\ 0 & H_m^{(1)}(k_2 r) & H_m^{(2)}(k_2 r) & -J_m(k_3 r) \\ 0 & \frac{k_2}{\mu_2} H_m^{(1)\prime}(k_2 r) & \frac{k_2}{\mu_2} H_m^{(2)\prime}(k_2 r) & -\frac{k_3}{\mu_3} J_m'(k_3 r) \end{vmatrix}$$

and D_m is

$$\begin{vmatrix} H_m^{(1)}(k_1 R) & -H_m^{(1)}(k_2 R) & -H_m^{(2)}(k_2 R) & 0 \\ \frac{k_1}{\mu_1} H_m^{(1)\prime}(k_1 R) & -\frac{k_2}{\mu_2} H_m^{(1)\prime}(k_2 R) & -\frac{k_2}{\mu_2} H_m^{(2)\prime}(k_2 R) & 0 \\ 0 & H_m^{(1)}(k_2 r) & H_m^{(2)}(k_2 r) & -J_m(k_3 r) \\ 0 & \frac{k_2}{\mu_2} H_m^{(1)\prime}(k_2 r) & \frac{k_2}{\mu_2} H_m^{(2)\prime}(k_2 r) & -\frac{k_3}{\mu_3} J_m'(k_3 r) \end{vmatrix}$$

Therefore, the normalized scattering and absorption cross sections could be calculated by following the formula [4]

$$Q_{\mathrm{scs}} = \frac{2}{k_1 R} \sum_{m=-\infty}^{\infty} |A_m|^2 \tag{S13a}$$

$$Q_{\mathrm{abs}} = -\frac{2}{k_1 R} \sum_{m=-\infty}^{\infty} \mathrm{Re}(A_m) - \frac{2}{k_1 R} \sum_{m=-\infty}^{\infty} |A_m|^2 \tag{S13b}$$

S2. Electrical Model for Hot-Electron Microprocesses

Hot-electron generation is a quantum process and in the region of existence time of femtoseconds [5], giving rise to non-equilibrium electrons whose energy distributions deviate substantially from equilibrium Fermi-Dirac distributions. The initial hot-electron energy distribution $D(E_e)$ is evaluated by [2]

$$D(E_{\mathrm{e}}) = \frac{S(E_{\mathrm{f}} - hv) f(E_{\mathrm{f}} - hv) S(E_{\mathrm{f}})[1 - f(E_{\mathrm{f}})]}{\int S(E_{\mathrm{f}} - hv) f(E_{\mathrm{f}} - hv) S(E_{\mathrm{f}})[1 - f(E_{\mathrm{f}})] \mathrm{d}E_{\mathrm{f}}} \tag{S14}$$

where $E_f = E_e + E_F$ is the full energy, hv the photon energy, $S(E_f)$ the parabolic electronic state density of free electron, and $f(E_f)$ the Fermi distribution function. Using Equation S14, the flux (N_{gen}) of generated hot electrons can calculated as

$$N_{\text{gen}} = \frac{I_{\text{in}} \times Q_{\text{abs}} \times 2 \times R}{h\nu} \quad \text{(S15)}$$

The generated hot electron would diffuse to the Ag–TiO_2 interface, undergoing transport losses due to electron-electron and electron-photon scatterings. In the framework of the designed coaxial structure, transport probability (P_{tra}) are employed to describe the chance that a hot electron successfully reaches to the Ag–TiO_2 interface without losing energy. P_{tra} could be written as [6]

$$P_{\text{tra}} = \frac{\alpha(\rho,\varphi)}{2\pi} \int_0^{\varphi_{\max}} \frac{e^{-\frac{r_\Delta(\rho,\varphi)}{l_e}}}{\Delta\varphi} \mathrm{d}\varphi \quad \text{(S16)}$$

$$r_\Delta = \sqrt{\rho^2 + r^2 + 2r\rho\cos\varphi} \quad \text{(S17)}$$

$$\varphi_{\max} = \frac{\pi}{2} - \arcsin\frac{r}{r_\Delta} \quad \text{(S18)}$$

where $\alpha(\rho,\varphi)$ is the solid angle in cylinder coordinate system, l_e the electronic mean free path in Ag, and r_Δ the distance from the position where the hot electrons are generated to the Ag–TiO_2 interface, as shown in Figure S1. Based on P_{tra}, the flux (N_{tra}) of hot electrons arriving at the interface could be defined as

$$N_{\text{tra}} = \iiint N_{\text{gen}} \times P_{\text{tra}} \times D\mathrm{d}E_e \mathrm{d}x\mathrm{d}y \quad \text{(S19)}$$

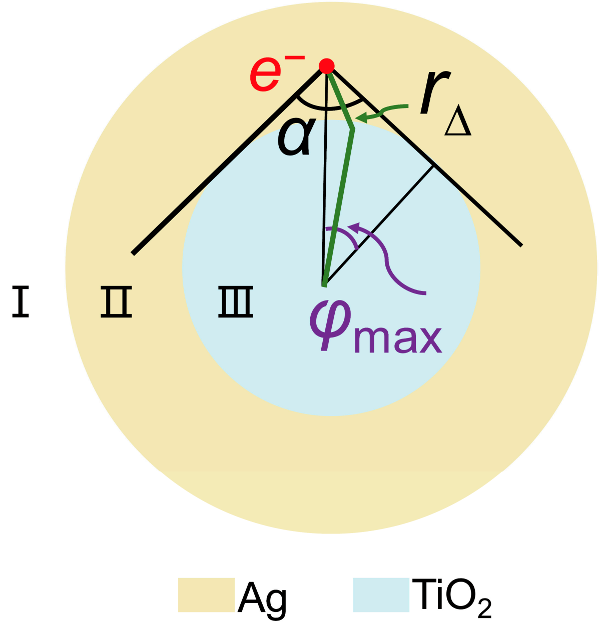

Figure S1. Device schematic and relevant parameters for evaluating hot-electron transport process.

Upon reaching the Ag–TiO_2 interface, the above-barrier hot electrons can be injected into TiO_2, suffering from E_e-dependent reflection losses. The hot-electron injection probability (P_{inj}) can be expressed as [7]

$$P_{\text{inj}}(E_e) = \int_0^{k_s} \frac{4\sqrt{k_m^2 - k_y^2} \times \sqrt{k_s^2 - k_y^2}}{(\sqrt{k_m^2 - k_y^2} + \sqrt{k_s^2 - k_y^2})} \times \frac{k_y}{2k_m\sqrt{k_m^2 - k_y^2}} \mathrm{d}k_y \quad \text{(S20)}$$

which k_y is the momentum components along the Ag–TiO_2 interface, k_m and k_s the momenta of hot electrons in Ag shell and TiO_2 core, respectively. k_m and k_s are defined as

$$k_m = \frac{\sqrt{2m_{\text{Ag}}E_e}}{\hbar} \quad \text{(S21a)}$$

$$k_{s}=\frac{\sqrt{2m_{TiO_2}(E_{e}-\Phi_{b})}}{\hbar} \tag{S21b}$$

In our study, m_{Ag} = 1.0m_0 and m_{TiO_2} = 0.9m_0 represent the effective mass of Ag and TiO_2, respectively [8]. m_0 is the mass of a free electron [9]. Finally, we obtain the flux (N_{inj}) of hot electrons transmitting across Schottky barrier [10]

$$N_{inj} = \iiint N_{gen} \times P_{tra} \times P_{inj} \times D\mathrm{d}x\mathrm{d}y\mathrm{d}E_{e} \tag{S22}$$

S3. Wavelength Dependence of IQE and EQE

Figure S2 shows the IQE of the 1st to 8th order Mie resonances as a function of wavelength in the range from 850 nm to 920 nm. For each resonance order, device IQE decreases with the increases of wavelength. At a fixed wavelength, the IQE is highest for the 1st order and lowest for the 8th order, indicating a clear dependence on the order $|m|$ and device size. Figure S3 shows the EQE of the 1st to 8th order Mie resonances as a function of wavelength from 850 to 920 nm. For each order, the variation of EQE with wavelength follows the same trend as the absorption efficiency, because EQE is proportional to the absorption efficiency in the Ag shell.

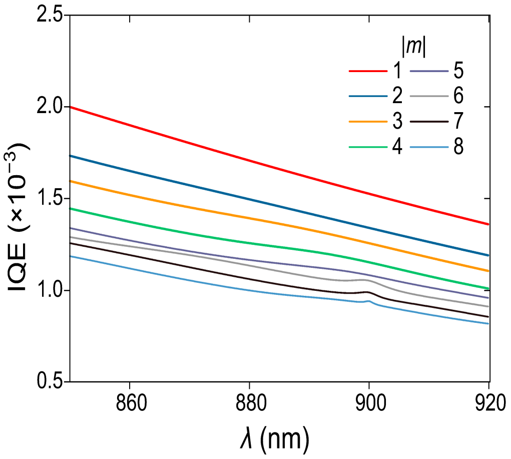

Figure S2. Wavelength dependence of IQE for the resonance orders 1st to 8th.

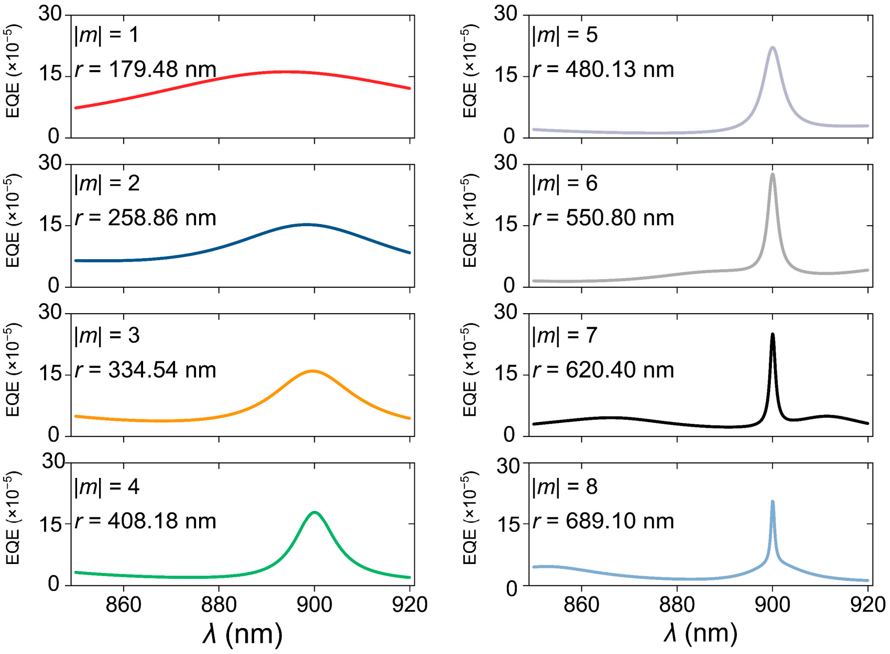

Figure S3. Wavelength-dependent EQEs for different $|m|$.

S4. Photoelectric Responses under TM-Polarized Incidence

To further investigate the coupled role of the resonance order and geometry in the designed SN HE-PD, we also considered a TM-polarized incident wave (i.e., magnetic field component oscillates along the *z* direction) illuminating the same Ag–TiO_2 coaxial nanowire. First, we calculated the Q_{abs} as a function of r and λ, as shown in Figure S4. Similar to the TE case discussed in the main text, resonances can still be excited at 900 nm by tuning the r while keeping the Ag shell thickness fixed at 8 nm. These TM-polarized resonances are presented in Figure S5. Unlike the TE-polarized case, only four resonances (the 1st, 6th, 7th, and 8th orders) are observed in the magnetic field distributions. These four orders exhibit independent resonances under TM polarization, while the other orders (2nd to 5th) are too weak to be clearly resolved in the simulations. Therefore, we only focus on the four strong resonances (the 1st, 6th, 7th, and 8th orders). As can be seen in Figure S6, the field confinement of TM modes is weaker than that of TE modes, leading to a lower Q_{abs} compared to the TE case discussed in the main text.

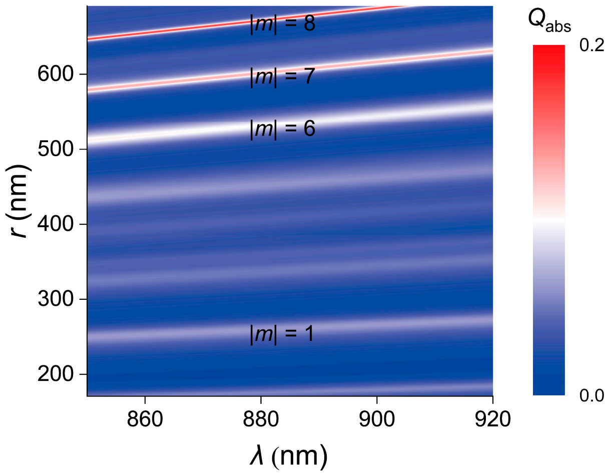

Figure S4. Q_{abs} change as with operating wavelength λ and the radius of TiO_2 r.

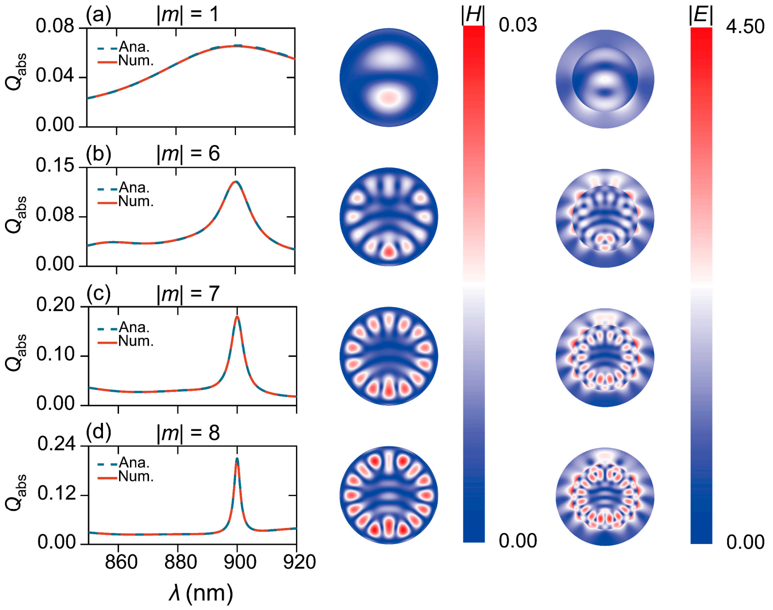

Figure S5. Excitation of TM-polarized Mie resonances of different orders at 900 nm by choosing an appropriate r. The left panels show the Q_{abs} as a function of λ.

The middle and right panels display the corresponding magnetic field and electric field distributions at the resonance peaks, respectively. The TiO_2 core radii are: (a)

266.25 nm, (b) 616.35 nm, (c) 688.05 nm, and (d) 758.35 nm, corresponding to the 1st, 6th, 7th, and 8th order Mie resonances, respectively.

After analyzing the optical characteristics of the designed hot-electron device, we now turn to electrical performance under TM-polarized illuminations. Figure S6 shows the device IQE for TM-excited resonances (1st, 6th, 7th, and 8th orders) over the wavelength range of 850–920 nm. The IQE decreases with increasing wavelength, similar to the trend in the case of TE-polarized excitation. We also present wavelength-dependent EQE in Figure S7. It is revealed EQE reaches the maximum at 900 nm and their full width at half maximum becomes small as the order increase. The EQEs for these orders are smaller than the main text because the optical absorption efficiency in Ag is smaller than that when TE-polarization is considered. Therefore, TE polarization is superior to TM polarization.

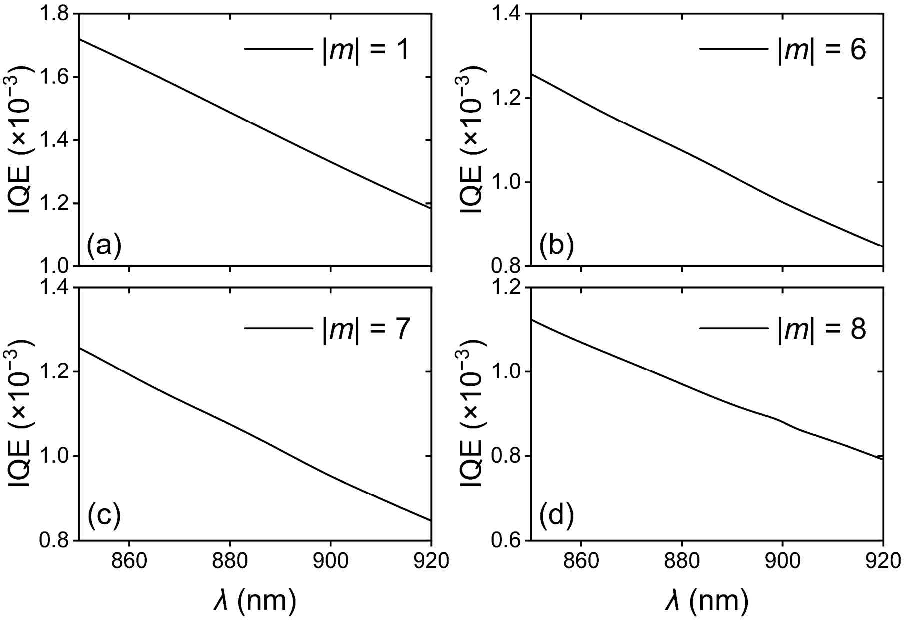

Figure S6. Wavelength-dependent IQE for the 1st, 6th, 7th, and 8th order resonances under TM polarization.

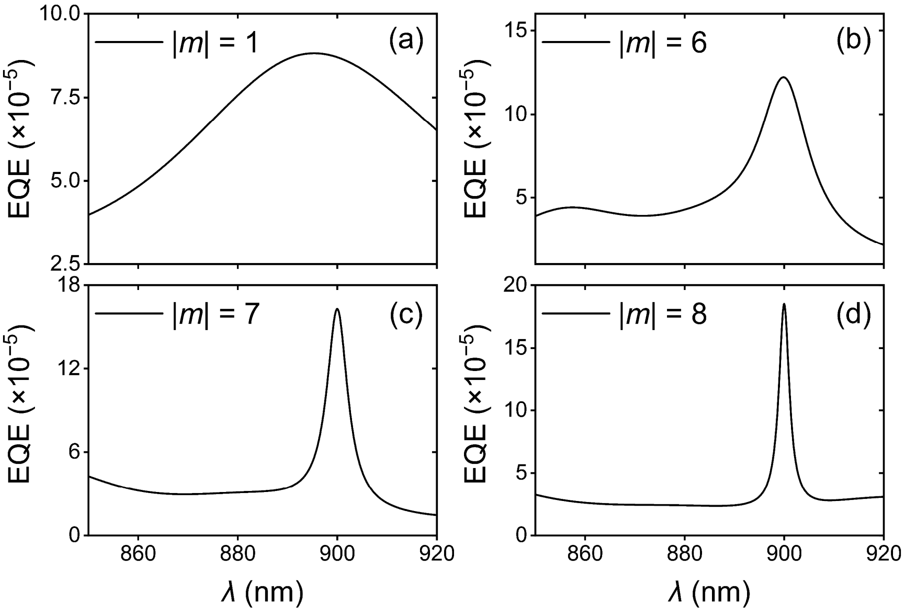

Figure S7. Wavelength-dependent EQE for the 1st, 6th, 7th, and 8th order resonances under TM polarization.

References

1. Toscano, G.; Raza, S.; Jauho, A.; et al. Modified field enhancement and extinction by plasmonic nanowire dimers due to nonlocal response. *Opt. Express.* 2012, 20, 4176–4188.
2. Zhang, C.; Wu, K.; Giannini, V.; et al. Planar hot-electron photodetection with Tamm plasmons. *ACS Nano*. 2017, 11, 1719–1727.
3. Zhou, Q.; Fu, Y.; Liu, J.; et al. Plasmonic bound states in the continuum in compact nanostructures. *Adv. Opt. Mater.* 2022, 10, 2201590.
4. Pors, A.; Moreno, E.; Martin-Moreno, L.; et al. Localized spoof plasmons arise while texturing closed surfaces. *Phys. Rev. Lett.* 2012, 108, 223905.
5. Khurgin, J. Fundamental limits of hot carrier injection from metal in nanoplasmonics. *Nanophotonics.* 2019, 9, 453–471.
6. Zhan, Y.; Li, X.; Wu, K.; et al. Coaxial Ag/ZnO/Ag nanowire for highly sensitive hot-electron photodetection. *Appl. Phys. Lett.* 2015, 106, 081109.
7. Chalabi, H.; Schoen, D.; Brongersma, M.; et al. Hot-electron photodetection with a plasmonic nanostripe antenna. *Nano Lett.* 2014, 14, 1374–1380.
8. Roh, Y.; Jin, Y.; Jeon, B.; et al. Revealing the loss mechanism of chemically-induced hot electron transport. *Nano Lett.* 2024, 24, 3490–3497.
9. Li, W.; Valentine, J. Harvesting the loss: surface plasmon-based hot electron photodetection. *Nanophotonics.* 2016, 6, 177–191.
10. Shao, W.; Li, Y.; Hao, J.; et al. Ultra-narrowband hot-electron photodetection with Friedrich-Wintgen bound states in the continuum. *Opt. Express.* 2026, 34, 16622–16636.
